# Chronic cough due to laryngeal hamartoma: A case report

**DOI:** 10.1016/j.ijscr.2025.111349

**Published:** 2025-04-23

**Authors:** Majd Oweidat, Eshraq Shalalfa, Munther Suleiman Abdalah Atawneh, Mohammed Alra'e, Yousef Abu Asbeh, Motaz Natsheh

**Affiliations:** a College of Medicine, Hebron University, Hebron, West Bank, Palestine; b Department of ENT, Halhul Governmental Hospital, Hebron, West Bank, Palestine; c Department of Thoracic Surgery, Al-Ahli Hospital, Hebron, West Bank, Palestine; d Department of Surgery, Al-Quds University, Jerusalem, Palestine; e Department of Pathology, Al-Ahli Hospital, Hebron, West Bank, Palestine

**Keywords:** Larynx, Laryngeal hamartoma, Bardet-biedl syndrome, Chronic cough, Histopathology, Case report

## Abstract

**Introduction:**

Laryngeal hamartomas (LHs) are rare, benign tumor-like growths arising from disorganized mature tissues. Bardet-Biedl syndrome (BBS) is a ciliopathy with multisystem manifestations. This article presents a rare case of LH presented with chronic cough.

**Presentation of case:**

A male in his 30s with BBS presented with a six-month history of persistent productive cough unresponsive to standard treatments. Video rhinolaryngoscopy and CT imaging identified a polyp on the anterior wall of the epiglottis. Histopathology confirmed the diagnosis of a hamartoma. Initial surgical excision was performed, preceded by a single IV dose of *Hydrocortisone*. Systemic corticosteroid therapy with oral *Prednisolone* tablets were prescribed postoperatively. Despite initial symptom resolution, the lesion recurred, necessitating re-excision and cautery. Histopathology suggested an inflammatory reaction rather than true recurrence.

**Discussion:**

LHs are uncommon, with fewer than 35 cases documented. They often mimic other laryngeal lesions, making diagnosis reliant on histopathological evaluation. This case marks the first reported instance of LH in a BBS patient. Management approaches, including careful surgical excision and post-operative care, are crucial to preserving laryngeal function.

**Conclusion:**

LH is a rare but important consideration in cases of chronic cough, especially when common causes have been ruled out.

## Highlights

• Rare case of chronic cough caused by laryngeal hamartoma (LH)
• Histopathological findings differentiated recurrence from inflammatory reaction post-surgery.
• Successful management involved excision, monopolar cautery, and tailored follow-up care with laryngoscopy and imaging.

## 1 Introduction

A hamartoma is a benign tumor characterized by the abnormal growth of tissues native to a specific anatomical location in the body. These masses can develop in various parts of the body, with the liver, spleen, gastrointestinal tract, pancreas, and kidneys being the most frequently involved organs [1,2]. It has also been reported in the nasosinusal area, nasopharynx, oral cavity, hypopharynx, esophagus, and larynx; however, its occurrence in the head and neck regions is rare [3]. In many cases, these lesions are asymptomatic and are typically found incidentally during the investigation of other conditions. However, complications, including those resulting from mass effects or even malignancy, can develop in some instances [2,4].

Bardet-Biedl syndrome (BBS) is an autosomal recessive disorder, characterized by multisystem ciliopathy, with wide range of manifestations, such as rod-cone dystrophy in the retinae, obesity, polydactyly, intellectual disabilities, renal abnormalities, hypogonadism, and other anomalies [5].

Herein, we report a rare case of LH presented with chronic cough.

The work has been reported in line with the SCARE Guidelines 2023 criteria [6].

## 2 Presentation of case

A known case of BBS male patient in his 30s, presented to our clinic complaining of a persistent cough lasting approximately six months. The cough was productive, episodic, and characterized by mucopurulent sputum. The patient denied experiencing fever, headache, hemoptysis, difficulty breathing (both during the day and at night), sneezing, stridor, wheezing, or any changes in voice tone. A comprehensive systemic review revealed no additional symptoms of concern.

The patient's medical history was significant for chronic renal impairment, blindness, infertility, and delayed mental abilities, all associated with his underlying genetic condition. Despite being treated for upper and lower respiratory tract infections over an extended period, his symptoms showed no improvement.

On physical examination, the patient was conscious, oriented, and appeared to be in good general condition. His mucosal color was normal, with no signs of pallor or cyanosis. Examination of the oral cavity showed normal mucosal color, and there were no indications of acute oropharyngitis. Bilateral nasal cavities were patent. Chest auscultation revealed good air entry bilaterally, with no wheezes or crepitations. Cardiac examination showed a regular heart rhythm with no additional sounds.

Video rhinolaryngoscopy identified a polyp on the anterior wall of the epiglottis. The vocal cords were mobile bilaterally, and no other abnormalities were observed. Chest X-ray findings were unremarkable. A computed tomography (CT) scan of the neck and chest showed no significant abnormalities apart from a polyp placed as described by the video rhinolaryngoscopy. Additionally, a few anterior cervical lymph nodes were mildly enlarged, with no evidence of lung masses or pulmonary nodules.

The clinical impression suggested that the cough was reflexive, triggered by the polyp making contact with the surrounding structures. The patient was admitted and started on a regimen of *Cefazolin* (1 g) and a single preoperative dose of intravenous *Hydrocortisone* (100 mg), administered to reduce reflexive coughing and facilitate anesthesia induction, as the patient had a short neck, obesity, and was anticipated to be a difficult intubation case.

**Fig. 1:**
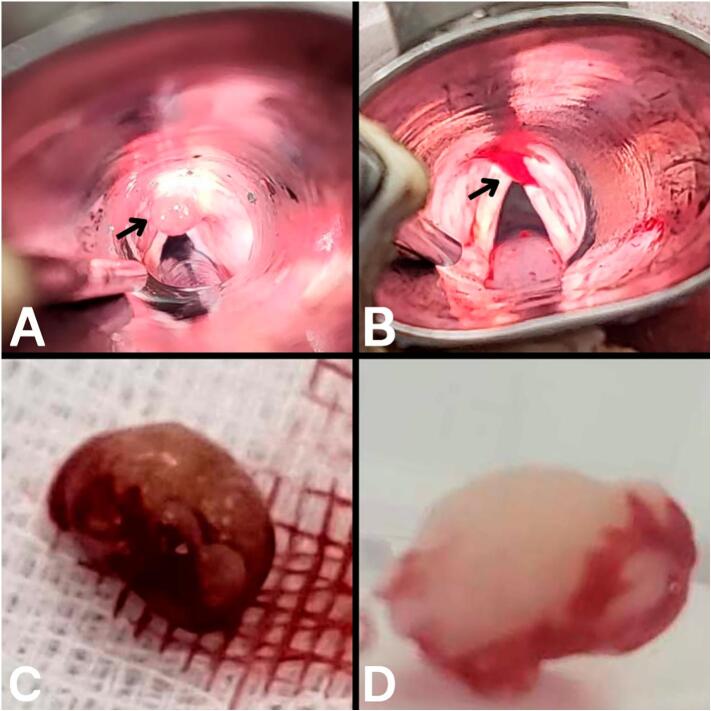
Intraoperative and postoperative imaging of the LH. A: Direct laryngoscopy showing the lesion (black arrow) on the anterior wall of the epiglottis wall before excision. B: Direct laryngoscopy demonstrating the successful removal of the lesion (black arrow). C: The excised lesion, shown grossly on a white gauze during the surgery. D: The excised lesion stored in formalin for histopathological examination.

**Fig. 2:**
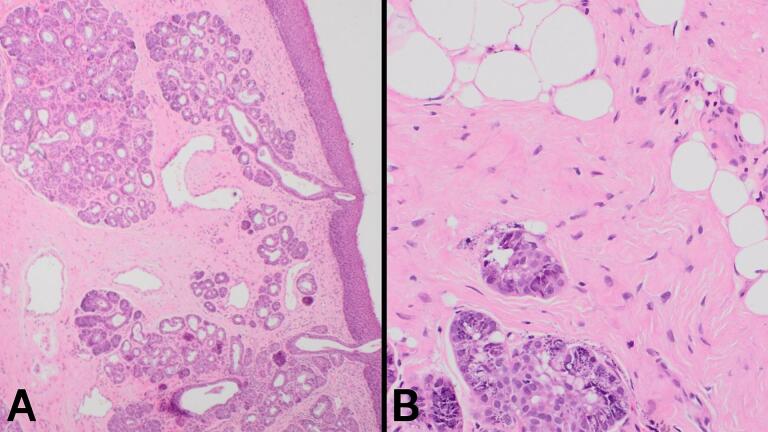
Histopathological examination of the first laryngeal lesion. A: Low magnification (H&E, 4×) revealed a polypoidal lesion composed of a mixture of benign mucus glands, benign adipose tissue, and disorganized blood vessels, all covered by respiratory epithelium. These findings are consistent with a diagnosis of LH. No evidence of dysplasia or malignancy was identified. B: Higher magnification (H&E, 20×) provides a detailed view of the benign tissue components.

The next day, a direct laryngoscopy was performed, during which the polyp was excised as shown in Fig. 1. The mass was removed via local excision, preserving the anterior wall of the epiglottis, and was subsequently stored in formalin. The sample was sent for histopathological examination, which revealed a polypoidal lesion with benign mucus glands, adipose tissue, and disorganized vessels, consistent with the diagnosis of a hamartoma (Fig. 2). After surgery, the patient was discharged home with a short course of Prednisolone tablets (2 mg daily for three days) to prevent upper airway edema following the manipulation and cautery performed during the procedure.

**Fig. 3:**
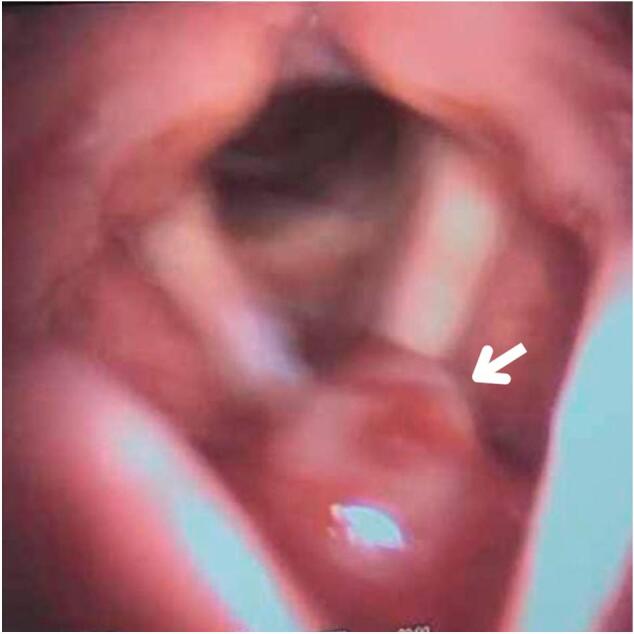
Video rhinolaryngoscopy five months post-surgery shows a recurrent lesion (white arrow) extends to the anterior commissure of the vocal cords, interfering with cord closure, and causing compression over the anterior commissure.

At a follow-up visit two weeks post-surgery, video rhinolaryngoscopy revealed a recurrent lesion with a broader base and involvement of the anterior commissure of the vocal cords. The anterior supraglottic wall examination was normal, with no signs of bleeding at the lesion site. However, during subsequent monthly follow-ups, the lesion gradually increased in size and extension, although the patient remained asymptomatic. Five months after the initial surgery, the patient returned with a recurrence of a more intense cough. At this point, the lesion had reached the anterior commissure of the vocal cords, interfering with cord closure as shown in Fig. 3.

The patient underwent another direct laryngoscopy and bronchoscopy, during which the recurrent polyp was removed using hot snaring, and the base was cauterized with monopolar electrical cautery. Intraoperatively, it was challenging to control the lesion, as it was not a true mass but rather a raised mucosal area with a wide basal extension, causing complete compression over the anterior commissure of the vocal cords. Care was taken to ensure that the anterior commissure was disengaged and moved freely following the procedure. The lesion was sent to pathology in multiple fragments. Histopathological examination revealed benign squamous epithelium with squamous hyperplasia, active inflammation with surface ulceration, and fibrotic stroma. These findings raised the suspicion that the lesion was not a true recurrence but rather a severe inflammatory reaction following the prior mass excision.

Post-operatively, the patient showed significant improvement. He was discharged home on a short course of *Prednisolone* tablets (2 mg daily for three days) to prevent upper airway edema following the surgery. Further follow-ups revealed no recurrence of the lesion on direct laryngoscopy and magnetic resonance imaging (MRI), nor did the patient experience any related symptoms. The vocal cords appeared healthy, with normal mobility and no evidence of residual inflammation or structural abnormalities. The patient remained asymptomatic, with no signs of recurrence during subsequent evaluations.

## 3 Discussion

Hamartomas are submucosal masses typically lacking encapsulation and having poorly defined borders [7]. These lesions result from disorganized growth of mature tissue, including epithelial or mesenchymal components. Mesenchymal hamartomas lack epithelial tissue, while glandular or epithelial hamartomas feature a mix of epithelial/glandular and mesodermal elements [7]. LHs are exceedingly uncommon. To date, fewer than 35 cases have been reported, with 22 occurring in pediatric patients [8].

LHs present similarly to other laryngeal lesions, with symptoms primarily determined by the lesion's location rather than its nature. A review by *Windfuhr JP* highlighted peak incidences of LHs in early childhood and between the ages of 50 and 60 years, with a male predominance (two-thirds of patients). The majority of hamartomas (around 70 %) are located in the supraglottic region [9]. The remaining cases occur in the subglottic area (8 %) and arytenoid/retrolaryngeal region (8 %). Symptoms vary based on the lesion's location and may include stridor, hoarseness, choking, dyspnea, and, in children, failure to thrive. Adults typically experience dyspnea, hoarseness, or dysphagia [9].

The diagnosis of LHs involves imaging modalities such as CT or MRI, with a definitive diagnosis established through histopathological examination. Histopathologically, LHs must be differentiated from other lesions, such as teratomas, choristomas, rhabdomyomas and dermoid cysts. Also malignant changes should be considered [3]. Malignant transformation of hamartomas with an unclear pathophysiology is rare but possible, as seen in Cowden syndrome, where risks of breast, thyroid, and endometrial cancers are increased [10]. Pulmonary nodules and gastrointestinal polyps in Peutz-Jeghers syndrome may also undergo malignancy. Breast hamartomas typically have the same malignant potential as normal breast tissue [10].

According to the literature, the management of LHs focused on complete surgical removal to restore optimal laryngeal function, including voice, airway, and swallowing [11]. Smaller, encapsulated lesions are typically excised endoscopically, while larger tumors may require more invasive approaches, including partial or total laryngectomy when preserving adequate laryngeal structure is unfeasible [12,13]. The prognosis for LHs is generally favorable, especially after complete excision, with minimal impact on the larynx function [9]. However, recurrence has been documented in 20 % of cases and is typically linked to the incomplete removal of the lesion [14]. Regular follow-ups with laryngoscopy and MRI are crucial for early detection of recurrence [9].

This case is the first reported instance of LH in a patient with BBS. Notably, only one case of false recurrence has been described—a 43-year-old female with vocal cord hamartoma, which was re-excised and found to be a scar rather than true recurrence [15]. Another case involved a two-month-old boy with congenital stridor and a cervical LH, which recurred post-excision, leading to fatal airway obstruction [16]. A 49-year-old male with chronic hoarseness underwent successful excision of a well-circumscribed laryngeal mass, with no recurrence after four years [17]. Similarly, a 4-year-old boy with progressive hoarseness had a laryngeal tumor excised after worsening symptoms. Histopathology confirmed hamartoma, and long-term follow-up showed no recurrence or functional impairment [18].

This case contributes to the limited literature on LH and shows the need to consider hamartomas in the evaluation of chronic cough. It also highlights that post-surgical inflammatory reactions may mimic true recurrence.

## 4 Conclusion

This case draws attention to LH as an uncommon but noteworthy cause of chronic cough. It deserves inclusion in the differential diagnosis when typical causes are have been ruled out. Further research to study the LHs is warranted.

## CRediT authorship contribution statement

MO: Conceptualization, data curation, writing – original draft preparation, writing – review & editing, investigation, visualization, validation, software and resources.

ES: Project administration, supervision, writing – original draft, methodology, and investigation.

MSAA: Methodology, investigation, reviewing and validation.

MA: Writing – original draft preparation, and data collection.

YA: Methodology, investigation, reviewing and validation.

MN: Methodology and investigation.

## Consent

Written informed consent was obtained from the patient for publication and any accompanying images. A copy of the written consent is available for review by the Editor-in-Chief of this journal on request.

## Ethical approval

Ethical approval was not applicable for this case, as the ethics committee at the *Department of ENT*, *Halhul Governmental Hospital*, *Hebron*, *Palestine*, does not mandate approval for reporting such individual cases.

## Guarantor

Eshraq Shalalfa

## Funding

None.

## Registration of research studies

Not applicable.

## Declaration of competing interest

None.

## Appendix A Supplementary data

Video 1 Indirect laryngoscopy visualizing the first lesion on the anterior wall of the epiglottis. Video 1

Video 2 Indirect laryngoscopy depicting the recurrent lesion on the anterior wall of the epiglottis five months post-surgery. The vocal cords are clearly visible and demonstrate movement. Video 2
